# Supplementary material for: Characterization of the Germplasm Bank for the Spanish Autochthonous Bull Breed “Asturiana de la Montaña”
Source: Animals (Basel). 2023 Apr 19;13(8):1402. doi: 10.3390/ani13081402 (PMC10135365; doi:10.3390/ani13081402)
Supplement: Supplementary file 1 [file animals-13-01402-s001.zip › animals-2289623-supplementary.pdf]

animals

MDPI

Supplementary material

Characterization of the germplasm bank for the Spanish autochthonous bull breed "Asturiana de la Montaña"

C. Tamargo, Amer Salman, J. Néstor Caamaño, Felipe Martínez-Pastor, A. Fernández, R. Muiño, M. T. Carbajo and Carlos O. Hidalgo

Table S1. Descriptive statistics from the fresh semen analysis (40 Asturiana de la Montaña bulls, 102 ejaculates).

Variable	Min.	1st Q.	Median	3rd Q.	Max.
Total motility (%)	65.5	83.9	93.4	95.9	98.5
Progressivity (%)	57.6	64.8	71.9	83.5	92.3
VCL (µm/s)	95.7	109.9	116.4	128.0	156.2
VAP (µm/s)	65.2	80.6	91.9	101.7	137
VSL (µm/s)	50.2	65.4	75.4	79.8	118.9
LIN (%)	49.1	58.3	63.9	68.8	76.5
STR (%)	75.7	78.4	80.7	83.9	86.8
WOB (%)	64.9	71	80.7	83.7	88.1
Abnormal forms (%)	3.0	8.6	8.8	8.9	13.0
Cytoplasmic droplets (%)	0	4.0	5.8	7.4	18.0
Damaged acrosomes (%)	1.0	3.0	3.7	4.2	8.0
HOST (%)	49.0	58.0	68.0	77.5	85.0
Viability (%)	55.0	63.0	66.0	70.2	80.0

Min: Minimum; 1st Q.: First quartile of the distribution; 3st Q.: Third quartile; Max.: Maximum.

Table S2. Descriptive statistics from the evaluation of the thawed semen (40 Asturiana de la Montaña bulls, 102 ejaculates).

Variable	Min.	1st Q.	Median	3rd Q.	Max.
Total motility (%)	6.3	26.4	34.8	42.6	63.0
Progressivity (%)	3.1	14.2	21.6	28.2	53.2
VCL (µm/s)	42.5	68.0	90.7	127.0	192.6
VAP (µm/s)	26.4	45.0	72.2	114.9	154.6
VSL (µm/s)	20.8	40.3	57.6	72.8	121.2
LIN (%)	49.1	59.0	62.8	67.2	78.6
STR (%)	68.7	80.0	87.8	92.5	95.7
WOB (%)	58.0	66.1	80.8	89.4	93.4
Abnormal forms (%)	9.0	17.4	17.6	17.7	33.0

Variable	Min.	1st Q.	Median	3rd Q.	Max.
Cytoplasmic droplets (%)	1.0	3.4	3.5	3.6	7.0
Damaged acrosomes (%)	9.8	15.3	18.5	23.4	50.9
HOST (%)	32.0	47.7	50.0	52.5	66.0
Viability (%)	13.9	47.1	53.9	62.2	78.1

Min: Minimum; 1st Q.: First quartile of the distribution; 3st Q.: Third quartile; Max.: Maximum.
